# Nonlinear Associations Between Frailty and Medication Burden in Hospitalized Older Adults

**DOI:** 10.1111/ggi.70566

**Published:** 2026-06-10

**Authors:** Hiroyuki Umegaki, Hirotaka Nakashima, Fumihiko Mizokami, Yosuke Yamada, Kazuhisa Watanabe, Chisato Fujisawa, Hitoshi Komiya, Tomihiko Tajima, Shosuke Satake, Yasushi Takeya, Mitsutaka Yakabe

**Affiliations:** ^1^ Department of Community Healthcare and Geriatrics, Graduate School of Medicine Nagoya University Nagoya Japan; ^2^ Institute of Innovation for Future Society Nagoya University Graduate School of Medicine Nagoya Japan; ^3^ Department of Pharmacy National Center for Geriatrics and Gerontology Obu Japan; ^4^ Department of Education and Innovation Training for Pharmacy National Center for Geriatrics and Gerontology Obu Japan; ^5^ Department of Geriatric Medicine National Center for Geriatrics and Gerontology Obu Japan; ^6^ Division of Health Science, Graduate School of Medicine University of Osaka Osaka Japan; ^7^ Department of Geriatric Medicine University of Tokyo Hospital Tokyo Japan

**Keywords:** anticholinergic burden, deprescribing, polypharmacy, potentially inappropriate medications

## Abstract

**Aim:**

To examine how medication burden varies across frailty stages in hospitalized older adults, with a focus on potential nonlinear associations:

**Methods:**

We analyzed a multicenter cohort of hospitalized adults aged ≥ 65 years. Frailty was assessed using the preadmission Clinical Frailty Scale (CFS). Associations between CFS and total medication count, potentially inappropriate medication (PIM) count, and anticholinergic burden (Japanese Anticholinergic Risk Scale) were evaluated using restricted cubic spline models adjusted for age, sex, and Charlson Comorbidity Index. Primary analyses were conducted in participants with complete data (*n* = 1075).

**Results:**

CFS showed significant associations with all medication‐related indicators (all overall *p* < 0.05), with evidence of nonlinearity for medication count (*p* < 0.0001) and PIM count (*p* = 0.024). Medication burden increased with worsening frailty, with the steepest rise observed in moderate frailty, followed by attenuation in more severe frailty. Across all indicators, adjusted curves suggested a consistent transition around CFS 5–6. These patterns were independent of age, sex, and comorbidity burden.

**Conclusions:**

Medication burden is strongly and nonlinearly associated with frailty in hospitalized older adults. These stage‐dependent patterns suggest that moderate frailty may represent a window for targeted medication review and deprescribing, supporting frailty‐stratified approaches to medication management in acute care.

## Graphical abstract

Medication burden in hospitalized older adults—including total medications, potentially inappropriate medications, and anticholinergic burden—showed nonlinear associations with frailty, peaking at moderate frailty (CFS 5–6) before attenuating in advanced frailty. These stage‐dependent patterns highlight moderate frailty as a key window for targeted medication review and deprescribing.

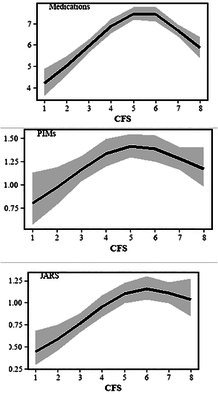

## 1 Introduction

Polypharmacy, exposure to potentially inappropriate medications (PIMs), and high anticholinergic burden are highly prevalent among hospitalized older adults and are associated with adverse drug events, delirium, functional decline, and mortality [1, 2]. Frailty, reflecting diminished physiological reserve and vulnerability to stressors, is a central construct in geriatric medicine and a major determinant of outcomes in acute care. Although frailty and medication burden frequently coexist, their relationship has often been assumed to be linear [3, 4, 5] This assumption may obscure clinically important stages at which pharmacologic risk is greatest.

Clinical experience suggests that prescribing patterns may vary across stages of frailty. In early frailty, accumulation of chronic diseases may increase medication use, whereas in advanced frailty therapeutic restraint or deprescribing may attenuate further increases in medication burden. However, empirical evidence supporting such stage‐dependent patterns in acute care settings remains limited.

The Clinical Frailty Scale (CFS) is widely used to stratify frailty severity in hospitalized patients and predicts mortality and functional decline [6, 7]. Understanding how medication burden varies across the CFS spectrum may clarify when older adults are most exposed to modifiable pharmacologic risk. We therefore examined associations between frailty and medication‐related indicators in hospitalized older adults, focusing on potential nonlinear relationships.

## 2 Methods

### 2.1 Study Design and Participants

This multicenter observational cohort study (J‐HAC study) was conducted at four acute care hospitals in Japan between October 2019 and March 2025 [8]. The study protocol was approved by the Ethics Committee of Nagoya University Graduate School of Medicine (approval no. 2019‐0260) and the institutional review boards of all participating centers. Written informed consent was obtained from all participants or their legal representatives in accordance with the Declaration of Helsinki.

Baseline information, including demographics, comorbidities, frailty, and admission medication profiles, was collected. A total of 1599 older patients were initially included in the registry. Of these, 492 were excluded because medication data at admission were missing. Among the remaining 1107 patients, 32 were excluded because of missing covariate data and/or frailty data or CFS = 9 because prescribing patterns in terminal illness are qualitatively different and primarily driven by end‐of‐life care. The final analytic cohort therefore comprised 1075 patients (Figure S1).

### 2.2 Frailty Assessment

Frailty was assessed using the preadmission CFS, scored from 1 (very fit) to 9 (terminally ill), based on health and functional status in the 2 weeks preceding hospitalization [9]. CFS was assessed by trained physicians or research staff at each participating hospital using information obtained from patients, family members, caregivers, and medical records. Investigators across sites shared standardized assessment procedures [10].

### 2.3 Medication‐Related Indicators

Medication burden at admission was characterized using three complementary indicators: (1) total medication count, (2) PIM count, and (3) anticholinergic burden measured by the Japanese Anticholinergic Risk Scale (JARS) [11]. Medication burden was assessed using medications already prescribed at the time of admission, reflecting prehospital pharmacotherapy. Regularly prescribed systemic medications were counted. As‐needed medications and topical agents were excluded unless administered regularly. Fixed‐dose combination drugs were counted according to the number of pharmacologically active components.

PIMs were defined according to the Guidelines for Medical Treatment and its Safety in the Elderly published by the Japan Geriatrics Society [12].

### 2.4 Covariates

Models were adjusted for age, sex, and Charlson Comorbidity Index (CCI) as markers of demographic and disease burden [13].

### 2.5 Statistical Analysis

Baseline characteristics were summarized across CFS categories. Nonlinear associations were examined using ordinary least squares regression models incorporating restricted cubic splines for CFS with four degrees of freedom. Internal knots were placed at the 25th, 50th, and 75th percentiles of the observed CFS distribution (CFS = 3, 5, and 6), with boundary knots at CFS = 1 and 8. Separate models were fitted for each medication indicator, and all models were adjusted for age, sex, and CCI. Robust standard errors were used to account for potential heteroskedasticity.

Overall and nonlinear components of the association between CFS and each outcome were evaluated using Wald statistics. Adjusted spline curves with 95% confidence intervals were generated. Peak locations along the CFS continuum were explored descriptively.

Although medication count and PIM count are discrete variables, preliminary assessment indicated approximately symmetric distributions with moderate‐to‐large means (mean medication count = 6.41). Because our primary objective was to characterize and visualize stage‐dependent nonlinear patterns, spline‐based linear models were used for the primary analysis.

Several sensitivity analyses were performed to assess robustness. First, negative binomial regression models were applied for medication and PIM counts to account for the count nature and potential overdispersion of the data. Second, CFS was modeled categorically (1–3, 4–5, 6–7, and 8) to examine whether similar patterns were observed without assuming continuity. Third, to address missing outcome data, inverse probability–weighted analyses were conducted, in which the probability of having medication data was modeled as a function of CFS, age, sex, and CCI, and weighted regression models were fitted using these weights. Additional sensitivity analyses were performed for PIM count and anticholinergic burden using quasi‐Poisson spline models to evaluate the robustness of the observed associations across different medication‐related indicators.

All statistical analyses were performed using R version 4.5.2 (R Foundation for Statistical Computing, Vienna, Austria). Two‐sided *p* < 0.05 were considered statistically significant.

## 3 Results

The analytic cohort comprised 1075 hospitalized older adults (median age 84 years, interquartile range [IQR] 80–88; 57.8% women). Baseline characteristics stratified by frailty category are shown in Table 1. With increasing frailty, participants were older, more likely to be female, and had a higher comorbidity burden. Median medication count increased from mild to moderate frailty and then attenuated in severe frailty. Similarly, the distributions of PIMs and anticholinergic risk scores shifted toward higher values with increasing frailty.

**TABLE 1:** Baseline characteristics of study participants stratified by clinical frailty scale score.

Characteristic	Overall (*N* = 1075)	CFS 1–4 (*N* = 387)	CFS 5–6 (*N* = 412)	CFS 7–8 (*N* = 276)
Age, years	84.0 (80.0, 88.0)	81.0 (77.0, 85.0)	86.0 (82.0, 89.0)	87.0 (82.0, 91.0)
Sex				
Male	454 (42.2%)	193 (49.9%)	173 (42%)	88 (31.9%)
Female	621 (57.8%)	194 (50.1%)	239 (58%)	188 (68.1%)
CCI	2.0 (1.0, 3.0)	1.0 (1.0, 3.0)	2.0 (1.0, 4.0)	3.0 (1.0, 4.0)
Medication count	7.0 (4.0, 10.0)	6.0 (3.0, 9.0)	7.0 (5.0, 11.0)	7.0 (4.0, 10.0)
PIM count				
0	343 (31.9%)	143 (37.0%)	119 (28.9%)	81 (29.3%)
1	288 (26.8%)	112 (28.9%)	98 (23.8%)	78 (28.3%)
2	226 (21.0%)	65 (16.8%)	105 (25.5%)	56 (20.3%)
3	121 (11.3%)	38 (9.8%)	46 (11.2%)	37 (13.4%)
4	61 (5.7%)	19 (4.9%)	27 (6.6%)	15 (5.4%)
5	25 (2.3%)	7 (1.8%)	10 (2.4%)	8 (2.9%)
6	6 (0.6%)	1 (0.3%)	5 (1.2%)	0 (0.0%)
7	3 (0.3%)	1 (0.3%)	1 (0.2%)	1 (0.4%)
JARS				
0	505 (47.0%)	201 (51.9%)	185 (44.9%)	119 (43.1%)
1	291 (27.1%)	107 (27.6%)	106 (25.7%)	78 (28.3%)
2	137 (12.7%)	41 (10.6%)	54 (13.1%)	42 (15.2%)
3	68 (6.3%)	21 (5.4%)	31 (7.5%)	16 (5.8%)
4	42 (3.9%)	11 (2.8%)	18 (4.4%)	13 (4.7%)
5	22 (2.0%)	3 (0.8%)	14 (3.4%)	5 (1.8%)
6	6 (0.6%)	1 (0.3%)	3 (0.7%)	2 (0.7%)
7	2 (0.2%)	1 (0.3%)	0 (0.0%)	1 (0.4%)
9	2 (0.2%)	1 (0.3%)	1 (0.2%)	0 (0.0%)

*Note:* Data are presented as median (25th, 75th percentile) for continuous variables and *n* (%) for categorical variables.

Abbreviations: CCI, Charlson Comorbidity Index; CFS, Clinical Frailty Scale; JARS, Japanese Anticholinergic Risk Scale; PIM, Potentially Inappropriate Medication.

**FIGURE 1:**
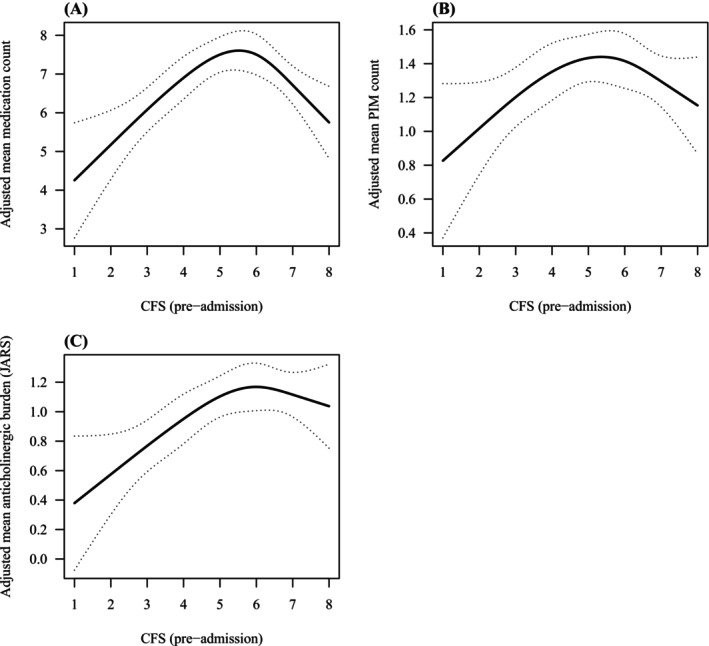
Restricted cubic spline curves showing adjusted associations between preadmission Clinical Frailty Scale (CFS 1–8) and (A) total medication count, (B) potentially inappropriate medication (PIM) count, and (C) anticholinergic burden (JARS) among 1075 participants with complete data.

Restricted cubic spline analyses demonstrated significant associations between CFS and all medication‐related indicators after adjustment for age, sex, and CCI. For total medication count, both the overall association and the nonlinear component were highly significant (both *p* < 0.0001) (Figure 1A). PIM count also showed a significant nonlinear association with CFS (overall *p* = 0.038; nonlinear *p* = 0.024) (Figure 1B). Anticholinergic burden was significantly associated with CFS (overall *p* = 0.0015), although evidence for nonlinearity was weaker and did not reach conventional statistical significance (nonlinear *p* = 0.068) (Figure 1C).

As shown in Figure 1, adjusted curves for all three indicators increased steeply from mild to moderate frailty and then attenuated in more severe frailty, with a consistent transition point around CFS 5–6. These associations were independent of chronological age, sex, and comorbidity burden.

Compared with participants with available medication data, those with missing medication data were slightly younger and less frail, while comorbidity burden was similar (median age 82 vs. 84 years; median CFS 4 vs. 5; median CCI 2 in both groups) (Table 2).

**TABLE 2:** Comparison of baseline characteristics between participants with and without available medication data.

Characteristic	Medication data available (*N* = 1107)	Medication data missing (*N* = 492)	*p*
Age, years	84.0 (80.0, 88.0)	82.0 (77.0, 86.5)	< 0.001
Sex			0.80
Male	462 (42%)	208 (42%)	
Female	645 (58%)	284 (58%)	
Preadmission clinical frailty scale			
1	18 (1.7%)	55 (12%)	
2	51 (4.7%)	42 (8.8%)	
3	146 (14%)	94 (20%)	
4	171 (16%)	88 (18%)	
5	183 (17%)	71 (15%)	
6	228 (21%)	73 (15%)	
7	227 (21%)	43 (9.0%)	
8	49 (4.5%)	8 (1.7%)	
9	5 (0.5%)	2 (0.4%)	
Unknown	29	16	
Charlson Comorbidity Index	2.0 (1.0, 3.0)	2.0 (1.0, 3.0)	> 0.90
Unknown	2	22	
Delirium			0.40
No	867 (83%)	404 (85%)	
Yes	175 (17%)	72 (15%)	
Unknown	65	16	
Falls during hospitalization			0.60
No	972 (94%)	449 (94%)	
Yes	67 (6.4%)	27 (5.7%)	
Unknown	68	16	
New pressure ulcer			0.062
No	1021 (98%)	461 (96%)	
Yes	22 (2.1%)	18 (3.8%)	
Unknown	64	13	
New urinary incontinence			0.50
No	1021 (98%)	465 (97%)	
Yes	20 (1.9%)	12 (2.5%)	
Unknown	66	15	
Hospital‐associated disability			0.010
No	726 (72%)	349 (78%)	
Yes	287 (28%)	98 (22%)	
Unknown	94	45	
Discharge outcome			
Death at 3 month after discharge	76 (6.9%)	25 (5.1%)	
Length of hospital stay, days	15.0 (9.0, 26.0)	12.0 (7.0, 23.0)	< 0.001

*Note:* Values are presented as median (Q1, Q3) or *n* (%). *p* values were calculated using the Wilcoxon rank‐sum test for continuous variables and Pearson's chi‐squared test for categorical variables.

Abbreviations: CCI, Charlson Comorbidity Index; CFS, Clinical Frailty Scale.

Sensitivity analyses demonstrated consistent findings across multiple analytical approaches. Similar nonlinear patterns with attenuation in severe frailty were observed in negative binomial models accounting for the count nature of medication data, inverse probability–weighted analyses (*n* = 1063) addressing missing medication data, and categorical CFS models that did not assume a continuous relationship (Table 3). Sensitivity analyses using quasi‐Poisson spline models demonstrated broadly similar associations for PIM count and anticholinergic burden. The association between frailty and PIM count remained significant (*p* < 0.001), and a similar overall association was also observed for anticholinergic burden (*p* < 0.001), supporting the robustness of the primary findings.

**TABLE 3:** Sensitivity analyses of the association between frailty and medication burden.

Model/sensitivity analysis	Population (*n*)	Outcome	CFS modeling	Main finding on CFS	*p* value for nonlinearity/overall CFS	Notes
Primary analysis (OLS + RCS)	*n* = **1075**	Medication count	Continuous (restricted cubic spline, df = 4)	Nonlinear increase with attenuation at higher CFS	**< 0.0001** (nonlinear)	CFS = 9 excluded; adjusted for age, sex, CCI
Negative binomial model	*n* = **1075**	Medication count	Continuous (restricted cubic spline, df = 4)	Similar nonlinear pattern preserved	***p* < 0.001** (overall CFS)	Count model sensitivity analysis
IPW analysis (missing medication data)	*n* = **1063**	Medication count	Continuous (restricted cubic spline, df = 4)	Nonlinear association preserved	***p* = 1.88 × 10** ^**−7**^ (overall CFS)	Weighted by probability of having medication data
Categorical CFS model	*n* = **1075**	Medication count	CFS 1–3, 4–5, 6–7, 8	Higher counts in CFS 4–7, attenuation at CFS 8	***p* < 0.001** (overall CFS)	Robust SE; adjusted for age, sex, CCI

*Note:* CFS was modeled using restricted cubic splines with four degrees of freedom, with internal knots at the 25th, 50th, and 75th percentiles (CFS = 3, 5, and 6) and boundary knots at CFS = 1 and 8. All models were adjusted for age, sex, and Charlson Comorbidity Index. Robust standard errors were used for linear models. Inverse probability weighting was applied to account for missing medication data. Patients with CFS = 9 were excluded from all primary and sensitivity analyses.

## 4 Discussion

In this multicenter cohort of hospitalized older adults, medication burden showed a robust nonlinear association with frailty severity. Medication count, PIM exposure, and anticholinergic burden increased from mild to moderate frailty but attenuated in severe frailty, with a transition around CFS 5–6.

These findings suggest that medication burden does not increase indefinitely with frailty. The steepest rise occurred in moderate frailty, when multimorbidity accumulates and disease‐oriented treatment strategies often intensify pharmacologic therapy. In contrast, attenuation in severe frailty may reflect therapeutic restraint, deprescribing, or shifts toward comfort‐oriented care. Survivor and selection effects may also contribute.

From a clinical perspective, the observed peak around CFS 5–6 may represent a transition point at which pharmacologic complexity and risk are greatest. These results extend prior work linking frailty and medication use [14, 15] by demonstrating that prescribing patterns vary across frailty stages rather than progressing in a simple monotonic fashion. Moderate frailty may therefore represent a particularly important window for structured medication review in acute care, when medication burden is highest and potentially modifiable. Incorporating frailty screening into routine hospital workflows could help clinicians identify patients most likely to benefit from proactive, interdisciplinary medication optimization and deprescribing efforts.

Importantly, the stabilization of overall medication burden in severe frailty should not be interpreted as evidence of appropriate medication optimization. In our study, both PIM exposure and anticholinergic burden showed trajectories broadly similar to total medication count, with no clear decline in high‐risk medications in advanced frailty. This pattern raises concern that prescribing in severe frailty may reflect global therapeutic restraint rather than systematic, goal‐concordant medication review [16]. Such nonspecific treatment attenuation may result not only in the persistence of potentially harmful drugs but also in the underuse of beneficial therapies, including symptom‐relieving, function‐supporting, or comfort‐oriented medications. In this context, severe frailty may be vulnerable not only to overtreatment but also to undertreatment, underscoring the need for individualized, frailty‐informed medication optimization rather than simple deprescribing. In contrast to medication count and PIM exposure, evidence for nonlinearity in anticholinergic burden was weaker. Although overall anticholinergic burden increased with frailty, the nonlinear component did not reach conventional statistical significance. This may suggest that anticholinergic exposure accumulates more gradually across frailty stages or reflects prescribing patterns that are less stage‐dependent than overall medication burden. Future studies examining specific medication classes across frailty stages may help clarify whether the observed attenuation in advanced frailty reflects selective deprescribing of preventive medications, continuation of symptom‐oriented therapies, or broader shifts in treatment goals.

Several limitations merit consideration. First, the observational design precludes causal inference, and residual confounding by unmeasured factors, such as disease severity, admission type, treatment goals, or clinician decision‐making processes, may have influenced prescribing patterns. Second, medication burden was assessed only at admission and did not capture dynamic changes during hospitalization, including deprescribing or treatment escalation, which may differ across frailty stages. Third, we did not directly assess medication appropriateness or clinical indication; therefore, the observed patterns cannot distinguish between appropriate, goal‐concordant prescribing and inappropriate polypharmacy or underprescribing. Fourth, a substantial proportion of participants lacked complete medication data. The specific reasons for missing medication data were not systematically recorded. Possible contributors include incomplete medication reconciliation at admission, transfer from outside facilities, and unavailable medication records. Although patients with missing medication data were younger and less frail, suggesting that the analytic cohort may represent a relatively more vulnerable population, selection bias cannot be excluded. However, inverse probability–weighted analyses yielded consistent results, supporting the robustness of the main findings. Fifth, survivor bias and treatment‐selection effects may partly explain the attenuation of medication burden in severe frailty, as patients with higher medication exposure may be less likely to survive or to be represented in the most advanced frailty categories. Differences in medication reconciliation practices and regional prescribing patterns across participating hospitals may have influenced the observed associations. Finally, as this study was conducted in hospitalized older adults in acute care settings, the generalizability of these findings to community‐dwelling populations or long‐term care settings may be limited.

In conclusion, medication burden in hospitalized older adults is strongly and nonlinearly associated with frailty, with a consistent attenuation in advanced frailty. These stage‐dependent patterns highlight moderate frailty as a potential target zone for intensified, frailty‐stratified, and risk‐focused medication review and support the integration of frailty assessment into acute care medication management strategies.

## Author Contributions

Study concept and design: Hiroyuki Umegaki. Acquisition of data: Hirotaka Nakashima, Fumihiko Mizokami, Yosuke Yamada, Kazuhisa Watanabe, Chisato Fujisawa, Hitoshi Komiya, Tomihiko Tajima, Shosuke Satake, Yasushi Takeya, and Mitsutaka Yakabe. Analysis and interpretation of data: Hiroyuki Umegaki. Drafting of the manuscript: Hiroyuki Umegaki. Critical revision of the manuscript for important intellectual content: All authors. Statistical analysis: Hiroyuki Umegaki. Administrative, technical, or material support: Hiroyuki Umegaki. Study supervision: Hiroyuki Umegaki.

## Funding

The authors have nothing to report.

## Disclosure

The authors have nothing to report.

## Conflicts of Interest

The authors declare no conflicts of interest.

## Supporting information


**Figure S1:** Study flow.

**Table S1:** Sensitivity analyses for potentially inappropriate medication (PIM) count and anticholinergic burden.

## Data Availability

The data that support the findings of this study are available on request from the corresponding author. The data are not publicly available due to privacy or ethical restrictions.
