# Supplementary material for: Nonlinear Associations Between Frailty and Medication Burden in Hospitalized Older Adults
Source: Geriatr Gerontol Int. 2026 Jun 10;26(6):e70566. doi: 10.1111/ggi.70566 (PMC13250668; doi:10.1111/ggi.70566)
Supplement: Supplementary file 1 — Figure S1: Study flow. [file GGI-26-0-s001.docx]

**Supplementary figure S1. Study flow**

Older adults aged ≥65 years enrolled in the multicenter J-HAC cohort

between October 2019 and March 2025

(N = 1,599)

Participants with available medication data

(medication count, PIM count, and JARS available) (N = 1,107)

Excluded from primary analytic cohort (N = 32)Missing covariate data and/or frailty data required for adjusted analyses or CFS = 9 (terminally ill)

Included in primary analytic cohort (N = 1,075)
