# Supplementary material for: Nonlinear Associations Between Frailty and Medication Burden in Hospitalized Older Adults
Source: Geriatr Gerontol Int. 2026 Jun 10;26(6):e70566. doi: 10.1111/ggi.70566 (PMC13250668; doi:10.1111/ggi.70566)
Supplement: Supplementary file 2 — Table S1: Sensitivity analyses for potentially inappropriate medication (PIM) count and anticholinergic burden. [file GGI-26-0-s002.docx]

**Supplementary Table S1 Sensitivity analyses for potentially inappropriate medication (PIM) count and anticholinergic burden**

| **Outcome** | **Sensitivity analysis** | **Population (n)** | **Main finding** | **P value** | **Notes** |
| --- | --- | --- | --- | --- | --- |
| PIM count | Quasi-Poisson spline model | 1,077 | Similar nonlinear association preserved across frailty stages | <0.001 (overall CFS) | Adjusted for age, sex, and Charlson Comorbidity Index |
| Anticholinergic burden (JARS) | Quasi-Poisson spline model | 1,080 | Overall association preserved, although evidence for nonlinearity was weaker | <0.001 (overall CFS) | Adjusted for age, sex, and Charlson Comorbidity Index |

Footnote:
CFS was modeled using restricted cubic splines with four degrees of freedom. Models were adjusted for age, sex, and Charlson Comorbidity Index. Quasi-Poisson regression models were used to account for the count nature and potential overdispersion of the outcomes.
